# Progress in Increasing Breastfeeding and Reducing Racial/Ethnic Differences — United States, 2000–2008 Births

**Published:** 2013-02-08

**Authors:** Jessica A. Allen, Ruowei Li, Kelley S. Scanlon, Cria G. Perrine, Jian Chen, Erika Odom, Carla Black

**Affiliations:** Div of Nutrition, Physical Activity, and Obesity, National Center for Chronic Disease Prevention and Health Promotion; Div of Blood Disorders, National Center on Birth Defects and Developmental Disabilities; Immunization Div, National Center for Immunization and Respiratory Diseases, CDC

The American Academy of Pediatrics recognizes breast-feeding and human milk as the “normative standards for infant feeding.” Given the documented health benefits, the Academy recommends exclusive breastfeeding for 6 months, followed by continued breastfeeding for at least 12 months as complementary foods are introduced (1). To better understand trends during 2000–2008 and differences in breastfeeding initiation and duration overall and among black, white, and Hispanic infants born in 2000 and 2008, CDC analyzed National Immunization Survey (NIS) data. Among infants born in 2000, 70.3% had ever breastfed (had breastfeeding initiated), 34.5% breastfed for 6 months, and 16.0% breastfed for 12 months. Among infants born in 2008, the comparable percentages had increased to 74.6%, 44.4%, and 23.4%, respectively. By race/ethnicity, prevalence of breastfeeding initiation in 2000 was 47.4% among blacks, 71.8% among whites, and 77.6% among Hispanics. By 2008, the percentage of infants who ever breastfed had increased among blacks to 58.9% and among whites to 75.2%; an 80.0% prevalence among Hispanics did not amount to a statistically significant increase. From 2000 to 2008, breastfeeding at 6 and 12 months increased significantly among all three racial/ethnic populations. Although the gap between black and white breastfeeding initiation narrowed, black infants still had the lowest prevalences of breastfeeding initiation and duration, highlighting the need for targeted interventions in this population to promote and support breastfeeding. Despite increases in the prevalence of breastfeeding, fewer than half of the infants in the survey were still breastfeeding at 6 months, indicating that women who choose to breastfeed their infants need support to continue breastfeeding.

NIS is an ongoing, random-digit–dialed telephone survey conducted quarterly in 50 states and the District of Columbia among households with children aged 19–35 months (2). The survey primarily is intended to estimate vaccination coverage nationally and by state and selected urban areas. However, questions on breastfeeding were added starting in the third quarter of survey year 2001, when a limited number of respondents were asked about breastfeeding. Beginning in January 2003, all respondents were asked breastfeeding questions. Interviews were conducted with the person in the house most knowledgeable about the eligible child’s vaccination history (2).

Because children are aged 19–35 months at the time of the NIS interview, each cross-sectional survey includes children born in earlier calendar years. For this report, a trend analysis for birth years 2000–2008 was conducted using data collected during 2002–2011. The data presented for infants born in 2000 were collected in 2002 and 2003; the data presented for infants born in 2008 were collected in 2009, 2010, and 2011. Breastfeeding initiation was assessed by asking, “Was [the child] ever breastfed or fed breast milk?” Breastfeeding duration was assessed by asking, “How long was [the child] breastfed or fed breast milk?” The wording of the breastfeeding duration question changed slightly in 2006 to “How old was [the child] when [the child] completely stopped breastfeeding or being fed breast milk?” These changes had minimal effect on estimates of breastfeeding duration (3).

The child’s race and ethnicity were reported by the respondent and categorized into one of three mutually exclusive racial/ethnic groups: white, black, and Hispanic. Persons identified as Hispanic might be of any race. Persons identified as white or black are non-Hispanic. The overall prevalences calculated included data from all racial/ethnic groups, not just the three included in this analysis.

Breastfeeding prevalences and 95% confidence intervals for each year were estimated as weighted percentages, taking into account the complex sampling design of NIS. Whether trends in breastfeeding percentages were statistically significant (p<0.05) during the 2000–2008 birth years was determined using polynomial linear contrasts. Additionally, for each year, the percentage of breastfeeding among black infants was compared with the percentages among white and Hispanic infants to ascertain significant differences by chi-square tests.

From 2000 to 2008, breastfeeding initiation increased overall from 70.3% to 74.6% (Table). Initiation increased from 71.8% to 75.2% among whites (p<0.01) and from 47.4% to 58.9% among blacks (p<0.01), but remained unchanged among Hispanics (77.6% to 80.0%, p=0.2). Breastfeeding duration at 6 months increased overall from 34.5% to 44.4%. Duration at 6 months increased from 38.2% to 46.6% among whites, 16.9% to 30.1% among blacks, and 34.6% to 45.2% among Hispanics (all p<0.01). Breastfeeding duration at 12 months increased overall from 16.0% to 23.4%. Duration at 12 months increased from 17.1% to 24.3% among whites (p<0.01), 6.3% to 12.5% among blacks (p<0.01), and 18.2% to 26.3% among Hispanics (p<0.01) (Table).

For each of the 2000–2008 birth years, breastfeeding initiation and duration prevalences were significantly lower among black infants compared with white and Hispanic infants. However, the gap between black and white breastfeeding initiation narrowed from 24.4 percentage points in 2000 to 16.3 percentage points in 2008 (Table).

## Editorial Note

The findings in this report indicate that from 2000 to 2008, significant increases occurred in the percentages of black and white infants who had ever breastfed, and in the percentages breastfeeding at 6 and 12 months among black, white, and Hispanic infants. However, although 74.6% of infants overall began breastfeeding in 2008, only 23.4% had the recommended duration of 12 months of breastfeeding (1). In addition, although differences might be decreasing between black infants and white and Hispanic infants, consistently lower prevalence of breastfeeding among black infants warrants increased attention and action.

A number of factors and characteristics influence a woman’s breastfeeding intentions. Characteristics associated with lower breastfeeding prevalence among women include younger age, lower income, less maternal education, and unmarried status (4). However, even when accounting for factors such as socioeconomic status and maternal education, racial/ethnic differences in breastfeeding persist (5,6). This persistent gap in breastfeeding rates between black women and women of other races and ethnicities might indicate that black women are more likely to encounter unsupportive cultural norms, perceptions that breastfeeding is inferior to formula feeding, lack of partner support, and an unsupportive work environment (7). All breastfeeding women need support, but specific interventions might be needed among populations with lower breastfeeding prevalence.

Although there is no single solution to increasing support for breastfeeding women, the 2011 *Surgeon General’s Call to Action to Support Breastfeeding* outlines a number of actions aimed at increasing societal support for women who choose to breastfeed (8). The report suggests that as communities, employers, health-care providers, governments, and nonprofit organizations implement strategies to support breastfeeding, all women who choose to breastfeed will benefit. Strategies to increase breastfeeding support for minority women include 1) increasing support for nonprofit organizations that promote breastfeeding in minority communities and 2) increasing the number of International Board Certified Lactation Consultants from minority communities (8).

CDC’s *Guide to Breastfeeding Interventions* also offers recommendations and program examples to assist states, territories, and communities in supporting mothers to begin and continue breastfeeding (9). Two projects currently funded by CDC aim to increase support for breastfeeding women by improving hospital practices related to breastfeeding and increasing community support available to breastfeeding women. The Best Fed Beginnings project provides support to 89 hospitals to improve maternity care practices to support breastfeeding women and to move the hospitals toward Baby-Friendly designation (10). Hospitals located in states known to have breastfeeding differences and serving low-income and minority populations were given preference. In addition, CDC awarded funds to six state health departments to develop community breastfeeding support systems in minority populations. Grantees will collaborate with community-based organizations to address the challenges that breastfeeding mothers encounter after hospital discharge to establish and maintain breastfeeding.

The findings in this report are subject to at least three limitations. First, the household response rates for NIS ranged from 61.6% to 74.2% during the survey years examined. Second, data collected before 2011 did not include cellular telephone users, introducing concern about how representative the data are for the population. However, sampling was adjusted for noncoverage of households without landline telephones. Although cellular telephone users were part of the 2011 survey, this analysis only includes the landline sampling frame, in order to allow comparison with previous years. Finally, the 2000 cohort is small compared with the other years because it includes only 2 years of survey data and in 2002 only a sample of respondents were asked breastfeeding questions.

What is already known on this topic?

The American Academy of Pediatrics recommends exclusive breastfeeding for 6 months, followed by continued breastfeeding for at least 12 months as complementary foods are introduced. Prevalences of breastfeeding initiation and duration have been increasing overall nationally. However, racial/ethnic differences in breastfeeding have been observed.

What is added by this report?

Trend analysis shows increases overall in U.S. breastfeeding from 2000 to 2008. The prevalence of infants ever breastfed increased from 70.3% to 74.6% during that period, breastfed at 6 months increased from 34.5% to 44.4%, and breastfed at 12 months increased from 16.0% to 23.4%. Breastfeeding prevalence among black infants was persistently lower than among whites and Hispanics. In 2008, prevalence of breastfeeding initiation was 58.9% among blacks, compared with 75.2% among whites and 80.0% among Hispanics.

What are the implications for public health practice?

Women who choose to breastfeed might need additional support to increase breastfeeding duration. A special need is for targeted strategies to increase breastfeeding support for black women.

The percentage of infants breastfeeding increased from 2000 to 2008. However, despite increases in the prevalence of infants ever breastfed and breastfeeding for 6 and 12 months, only a small percentage of infants were breastfed for the recommended minimum breastfeeding duration of 12 months, indicating that mothers might need support to continue breastfeeding. The prevalence of breastfeeding among black infants remains below that for whites and Hispanics, suggesting that black mothers might face unique barriers to meeting breastfeeding goals and might need additional support to start and continue breastfeeding.

## Figures and Tables

**TABLE:** Percentage of infants breastfed, by breastfeeding duration and race/ethnicity* — National Immunization Survey, United States, 2000 and 2008 births†

Duration and Race/Ethnicity	2000			2008			Percentage point increase from 2000 to 2008
	
	No. in sample	%	(95% CI)	No. in sample	%	(95% CI)	
**Ever breastfed**							
**Overall**§	**12,017**	**70.3**	**(68.4–72.3)**	**24,622**	**74.6**	**(73.6–75.5)**	**4.2**
White	6,631	71.8	(69.4–74.3)	15,119	75.2	(74.0–76.4)	3.4
Black	1,808	47.4	(41.4–53.4)	2,599	58.9	(56.1–61.8)	11.6
Hispanic	2,482	77.6	(73.6–81.7)	4,236	80.0	(78.0–81.9)	2.4¶
**Breastfed at 6 mos**							
**Overall**§	**12,017**	**34.5**	**(32.4–36.5)**	**24,622**	**44.4**	**(43.3–45.4)**	**9.9**
White	6,631	38.2	(35.5–40.9)	15,119	46.6	(45.3–47.9)	8.4
Black	1,808	16.9	(13.2–20.7)	2,599	30.1	(27.4–32.8)	13.2
Hispanic	2,482	34.6	(29.8–39.3)	4,236	45.2	(42.6–47.8)	10.7
**Breastfed at 12 mos**							
**Overall**§	**12,017**	**16.0**	**(14.4–17.6)**	**24,622**	**23.4**	**(22.5–24.4)**	**7.4**
White	6,631	17.1	(14.9–19.3)	15,119	24.3	(23.1–25.4)	7.2
Black	1,808	6.3	(4.0–8.7)	2,599	12.5	(10.5–14.4)	6.2
Hispanic	2,482	18.2	(14.2–22.1)	4,236	26.3	(23.7–28.8)	8.1

**Abbreviation:** CI = confidence interval.

* The child’s race and ethnicity were reported by the respondent and categorized into one of three mutually exclusive racial/ethnic groups: white, black, and Hispanic. Persons identified as Hispanic might be of any race. Persons identified as white or black are non-Hispanic.

† Data for 2000 and 2008 births were collected from survey years 2002, 2003 and 2010, 2011, 2012, respectively.

§ The overall values include data from all racial/ethnic groups, not just the three included in this analysis.

¶ Increase was not significant; all other increases presented in table were significant (p<0.05), based on trend analysis using polynomial contrasts.

## References

[1] American Academy of Pediatrics Section of Breastfeeding (2012). Policy statement: breastfeeding and the use of human milk. Pediatrics.

[2] CDC (2005). Statistical methodology of the National Immunization Survey, 1994–2002. Vital Health Stat.

[3] CDC (2012). Breastfeeding: NIS survey methods.

[4] Grummer Strawn L, Shealy K (2009). Progress in protecting, promoting, and supporting breastfeeding: 1984–2009. Breastfeeding Med.

[5] CDC (2006). Racial and socioeconomic disparities in breastfeeding—United States, 2004. MMWR.

[6] Li R, Grummer Strawn L (2002). Racial and ethnic disparities in breastfeeding among United States infants: Third National Health and Nutrition Examination Survey, 1988–1994. Birth.

[7] Ludington-Hoe S, McDonald PE, Satyshur R (2002). Breastfeeding in African-American women. J Natl Black Nurses Assoc.

[8] US Department of Health and Human Services (2011). The Surgeon General’s call to action to support breastfeeding.

[9] CDC (2012). The CDC guide to breastfeeding interventions.

[10] World Health Organization, United Nations Children’s Fund (2013). Baby-Friendly Hospital Initiative.

